# Studying the Defects in Spinel Compounds: Discovery, Formation Mechanisms, Classification, and Influence on Catalytic Properties

**DOI:** 10.3390/nano14201640

**Published:** 2024-10-12

**Authors:** Tetiana Tatarchuk

**Affiliations:** 1 Faculty of Chemistry, Jagiellonian University, ul. Gronostajowa 2, 30-387 Kraków, Poland; tatarchuk.tetyana@gmail.com; 2 Educational and Scientific Center of Materials Science and Nanotechnology, Vasyl Stefanyk Precarpathian National University, 76018 Ivano-Frankivsk, Ukraine

**Keywords:** defect, vacancy, spinel, ferrite, magnetism, structure

## Abstract

Spinel ferrites demonstrate extensive applications in different areas, like electrodes for electrochemical devices, gas sensors, catalysts, and magnetic adsorbents for environmentally important processes. However, defects in the real spinel structure can change the many physical and chemical properties of spinel ferrites. Although the number of defects in a crystal spinel lattice is small, their influence on the vast majority of physical properties could be really decisive. This review provides an overview of the structural characteristics of spinel compounds (e.g., CoFe_2_O_4_, NiFe_2_O_4_, ZnFe_2_O_4_, Fe_3_O_4_, γ–Fe_2_O_3_, Co_3_O_4_, Mn_3_O_4_, NiCo_2_O_4_, ZnCo_2_O_4_, Co_2_MnO_4_, etc.) and examines the influence of defects on their properties. Attention was paid to the classification (0D, 1D, 2D, and 3D defects), nomenclature, and the formation of point and surface defects in ferrites. An in-depth description of the defects responsible for the physicochemical properties and the methodologies employed for their determination are presented. DFT as the most common simulation approach is described in relation to modeling the point defects in spinel compounds. The significant influence of defect distribution on the magnetic interactions between cations, enhancing magnetic properties, is highlighted. The main defect-engineering strategies (direct synthesis and post-treatment) are described. An antistructural notation of active centers in spinel cobalt ferrite is presented. It is shown that the introduction of cations with different charges (e.g., Cu(I), Mn(II), Ce(III), or Ce(IV)) into the cobalt ferrite spinel matrix results in the formation of various point defects. The ability to predict the type of defects and their impact on material properties is the basis of defect engineering, which is currently an extremely promising direction in modern materials science.

## 1. Introduction

**Figure 1:**
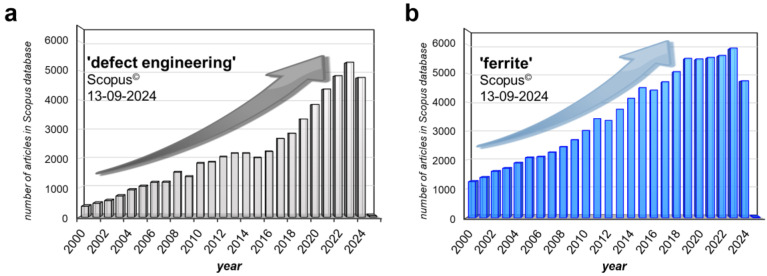
The number of published papers from 2000 to 2024 using the keywords (**a**) “defect engineering” and (**b**) “ferrite” indexed in the Scopus database (as of 13 September 2024).

Solid-state materials demonstrate a real structure that differs from the ideal (well-ordered) ones due to the presence of various defects. Some of them are formed during the synthesis (growth) of crystals (such as edge and screw dislocations, grain boundaries, pores, etc.), whereas others (intrinsic or impurity point defects) can be created during heat treatment [1,2]. All those defects (their types and concentrations) affect the properties of solid-state materials, and sometimes their influence is really significant [3]. Various important physical and chemical properties of materials (mechanical, optical, electrical, magnetic, catalytic, photocatalytic, adsorptive, etc.) strongly depend on the defects’ presence [4,5]. Hence, there has been a growing interest in the exploration of defects and the underlying mechanisms leading to their formation [6,7,8]. Over the past two decades, extensive research efforts have been dedicated to the investigation of defects (Figure 1a). Currently, the number of scientific publications dedicated to the studies on ‘defect engineering’, published in 2000–2024 years, and indexed in the Scopus database is more than 55,839 (as of 13 September 2024) (Figure 1a). This noticeable growth pattern can be attributed to the increasing research opportunities and advancements in laboratory techniques [9,10,11,12,13].

The migration of atoms in crystals can be carried out only if there are intrinsic defects (vacancies or interstitial atoms) or non-stoichiometric defects (heterovalent atoms or ions) [14,15]. The movement of atoms in the solids can be caused by the concentration gradient or external factors [16]. The migration of atoms due to the concentration gradient underlies such important processes as mass transfer and diffusion in solids. The diffusion is possible if the crystals have neutral or charged defects. An example of atom/ion migration due to the impact of external factors could be the migration of ions in crystals under the influence of an external electric field [17]. The movement of charged carriers in the crystal is the basis of ionic conductivity [18]. Ionic conductivity in crystals is possible only in the presence of charged defects [19]. Consequently, atomic defects in real crystals determine such important properties of solids as mass transfer, diffusion, and ionic conductivity. Similarly, the electronic conductivity of crystals (e.g., dielectrics and semiconductors) is impossible without the presence of electronic defects [20,21]. Therefore, it is not surprising that defect engineering of materials is a perspective direction in modern materials science [22,23,24]. The knowledge about various types of defects in crystals, mechanisms of their formation, and methods for control of defect formation are very important [25,26]. In this review, the main focus will be on defects that appear in spinel ferrite crystal lattice. Ferrites are compounds of iron(III) oxide with other metal oxides with both stoichiometric and non-stoichiometric composition. The magnetic, electrical, and physicochemical properties of ferrites are determined by the oxidation degree of metal ions, their distribution in the crystal lattice, and the presence of various types of defects. The controlled and rational processing of ferrite is pivotal in determining its structure and properties. Enhancing the controllability of ferrite particle processing poses a significant challenge, which can be addressed through various strategies. For example, the synthesis of nanocomposites, such as ferrite/polyaniline [27] or ferrite/graphene nanocomposites [28], offers a viable approach. In these instances, conducting polymers or carbon matrices play a crucial role in mitigating substantial agglomeration of magnetic ferrite nanoparticles, thereby amplifying their microwave absorption capacity. Consequently, these materials emerge as promising candidates for lightweight microwave absorbers.

Over the last twenty years, research on ferrites has gained much attention. The number of scientific publications dedicated to the studies on ferrites and published in 2000–2024 years is more than 88,600 in the Scopus database (as of 13 September 2024) (Figure 1b). The strategic management of defects in spinel compounds, including ferrites, is imperative for enhancing quality within the manufacturing industry, as it directly influences the material quality and desired physicochemical properties. The concentrations and types of defects, including vacancies, interstitials, dislocations, grain boundaries, and voids, significantly impact the functional properties of spinel compounds, playing a pivotal role in diverse applications, such as catalysis, adsorption, sensors, electrocatalysis, photocatalysis, microwave absorbance, and the development of optical and magnetic devices or magnetostrictive smart materials [4,29]. Even at relatively low concentrations, defects can wield a substantial influence on industrial processes, such as thin film growth. For instance, oxygen vacancies generated on the electrode surface play a critical role in augmenting the electrochemical performance of the electrode. Doped defects can serve as surface trappers for effectively segregating charge carriers and inhibiting the photocorrosion rate. Furthermore, the incorporation of vacancies in the spinel structure elevates electrical conductivity, fosters the establishment of surface active centers, and enhances intrinsic activity, consequently leading to improved electrochemical properties. In this review, the focus on examining how defects affect the properties of spinel ferrites will be made.

## 2. Spinel Compounds: Crystal Structure, Types, and Chemical Bonds

**Figure 2:**
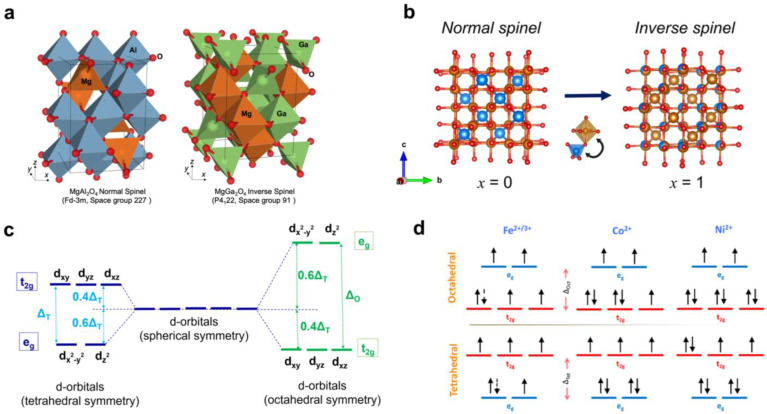
(**a**) Schematic illustration of the normal and inverse spinel structure (side view) (reprinted with permission from [38]. Copyright 2020 Springer Nature). (**b**) Top view of lattice cells of normal (**left**) and inverse (**right**) spinel ferrites (the oxygen ions are marked in red, Fe^3+^ ions are marked in orange, and M^2+^ ions are marked in blue) (reprinted with permission from [34]. Copyright 2021 American Chemical Society). (**c**) The d-orbitals splitting for the A- and B-cations in spinel sublattices. (**d**) The filling of the t_2g_ and e_g_ orbitals for the Fe, Co, and Ni cations (reprinted with permission from [15]. Copyright 2016 American Chemical Society).

Many widely used ferrites have a characteristic spinel structure, derived from a known natural mineral “spinel” with a general formula of MgAl_2_O_4_ [30]. The replacement of Al^3+^ with Fe^3+^ results in the ferrospinel formation [31]. Usually, the general formula of ferrospinels is MFe_2_O_4_, where M is a divalent cation (Co^2+^, Fe^2+^, Ni^2+^, Zn^2+^, Mg^2+^, Mn^2+^, etc., or a combination of two cations 0.5Li^+^ + 0.5Fe^3+^) [32]. The maghemite γ-Fe_2_O_3_ also has a spinel structure, which can be represented by the formula ⎕13Fe233+Fe23+O42–, where the symbol ⎕ means a cation vacancy. The lattice cell of ferrospinels is a densely packed face-centered cubic lattice of oxygen anions, and it contains 32 O^2−^ anions (Figure 2a). The 24 metal cations (8 A^2+^ cations and 16 B^3+^ cations) are located in the cavities formed by the oxygen anions. The A cations occupy tetrahedral sites formed by four oxygen anions, while the B cations occupy octahedral sites formed by six oxygen anions [33]. The crystallochemical formula of spinel ferrites can also be represented as (M^2+^_1−*x*_Fe^3+^*_x_*)_A_[M^2+^*_x_*Fe^3+^_2−*x*_]_B_O_4_, where *x* is the inversion parameter that shows the fraction of trivalent metal cations in the tetrahedral (A) positions; A and B are tetrahedral and octahedral sites, respectively [34].

There are four main types of spinel ferrites depending on the *x* value: (i) *x* = 0 means normal spinel, (ii) *x* = 1 means an inverse spinel, (iii) *x* = 2/3 points out a fully randomized spinel, and (iv) 0 < *x* < 1 means a mixed or partially inverted spinel [34] (Figure 2b). There are two non-equivalent crystallographic sublattices—tetrahedral (A–) and octahedral (B–)—in the spinel structure. If there are two or more cations at least in one sublattice, their arrangement (formation of superstructure) is possible, which leads to the appearance of new chemical and physical properties [35,36].

The distance between the centers of neighboring A–A cations, located at the A-sites, is approximately 0.365 nm, while the distance between neighboring B–B cations is approximately 0.297 nm. The distance between cations occupying A and B sites, respectively, is approximately 0.348 nm [33]. Substituting cations with different ionic radii disrupts the dense cubic packing of the spinel lattice.

The volume of the tetrahedral voids is subject to increase or decrease based on the radius of substituent ions, leading to the displacement of the surrounding four oxygen ions along the spatial diagonals of the cube. This results in the formation of expanded or compressed tetrahedra and the simultaneous distortion of octahedral voids. The displacement of oxygen ions is quantified by the anion parameter, denoted as *u* [37]. In an ideal scenario, *u* = 3/8 = 0.375; however, in real ferrites, *u* usually ranges from 0.380 to 0.385 [35,36]. This signifies that the oxygen parameter is defined by the formula *u* = 0.375±δ, where δ represents the deviation from the ideal anion parameter *u*. Given the value of the anion parameter, it is feasible to compute the interionic distances lA−O=3au−14 and lB−O=a2u−382+58−u212, where *a* denotes the lattice parameter [33]. Furthermore, the angle between bonds A–O–B is approximately 125°, while the angle between bonds B–O–B is around 90° [37].

Each cation in the A- and B-sublattice of the spinel structure is surrounded by four or six oxygen ions, respectively, which causes a repulsive effect known as Madelung potential [39]. This, in turn, causes the appearance of a crystal field effect and, as a consequence, the splitting of d-orbitals on t_2g_ and e_g_ orbitals (Figure 2c). As an example, the filling of the t_2g_ and e_g_ orbitals for the Fe, Co, and Ni cations is demonstrated in Figure 2d. The splitting of the d-orbitals of A- and B-cations affects the d-band by causing it to narrow. The interaction between the broad p-band of oxygen anions and the narrow d-band of cations through ionic and covalent interactions is quite significant. This interaction leads to the overlap between the electrons from cations and the oxygen orbitals, resulting in the formation of σ- and π-bonding molecular orbitals (MOs) and antibonding molecular orbitals (MO*) [39]. The available orbitals exist in both hybridized and non-hybridized states, ultimately leading to the formation of a 3D crystal band and enabling the construction of a density of states (DOS) diagram. The research [39] indicates that the position of the oxygen p-band center and the relative position between the oxygen p-band and the metal d-band play a significant and ongoing role in regulating the OER mechanism. This suggests that an interplay between these two bands significantly influences the catalytic reactions.

Moreover, the presence of octahedral BO_6_ units in spinel structure allows for the designing of great catalysts, for example, for OER processes [39]. At the same time, it is worth noting that the covalency competition between A- and B-sites (marked as M_T_–M_O_ competition) should be taken into account [39]. The overlap between the oxygen p-orbitals and cations d-orbitals results in asymmetrical M_T_–O–M_O_ bonds, in which either M_T_–O or M_O_–O is weaker [39]. In catalytic processes occurring within a redox environment, the breaking of weaker bonds may lead to the appearance of two unsaturated residual bonds, namely M–O and M−. It is observed that metal cations bound to oxygen anions do not significantly contribute to the reaction performance due to their fully saturated bonds [39]. Conversely, M−, characterized by unpaired electrons, holds the potential to serve as an active center by attracting OH−, thereby facilitating the generation of M–OH species critical for initiating catalytic processes [39]. Consequently, deliberate and precise adjustment of the cation composition has a notable impact on the covalency competition between A- and B-cations, leading to the formation of asymmetric M_O_–O–M_T_ bonds and the appearance of catalytically active sites on the spinel surface.

**Figure 3:**
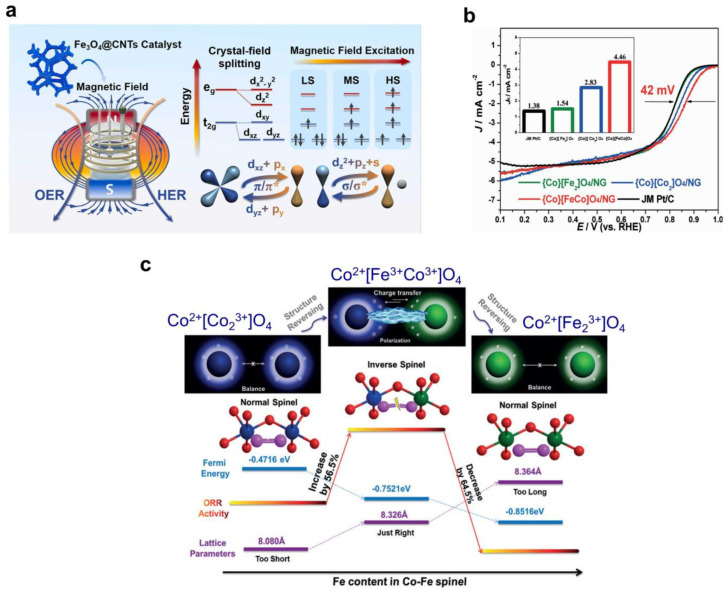
(**a**) The utilizing of the alternating magnetic field to induce the transition from ‘low-spin’ to ‘high-spin’ states in octahedral Fe ions in the Fe_3_O_4_@CNTs heterostructure (reprinted with permission from [40]. Copyright 2024 Elsevier). (**b**) The linear sweep voltammetry in an O_2_-saturated 0.1M KOH solution for {Co}[Fe_2_]O_4_/NG, {Co}[Co_2_]O_4_/NG, {Co}[FeCo]O_4_/NG, and Pt/C (reprinted with permission from ref. [41], copyright 2016 Wiley-VCH). (**c**) The dependence between the structure inversion and ORR activity for Co–Fe-based spinels (Fe green, Co blue, absorbed O magenta, lattice O red) (reprinted with permission from ref. [41], copyright 2016 Wiley).

In a study referenced as [40], an alternating magnetic field was utilized to induce the transition from ‘low-spin’ to ‘high-spin’ states in octahedral Fe ions in magnetite and Fe_3_O_4_@CNTs heterostructure (Figure 3a). This transition resulted in increased charge transfer of unpaired d-electrons, thus promoting interactions between σ- and π-d-orbitals between the intermediates and active sites. Consequently, an enhanced electrocatalytic activity was observed. Additionally, DOS analysis indicated that the d-band center serves as an indicator of the degree of d-orbital filling, consequently exerting a notable influence on the intrinsic catalytic activity of the catalyst [40].

It should be noted that the transformation of spinel structure from normal to inverse has a notable impact on the oxygen reduction reaction (ORR) activity of spinels [41,42]. For instance, the electrocatalytic performance of Co–Fe-based spinels exhibited significant enhancement following the transformation of their structure (as depicted in Figure 3b,c) [41]. The ORR activity of the inverse {Co}[FeCo]O_4_ spinel surpasses that of commercial Pt/C by 42 mV in an alkaline medium (Figure 3b). The DFT calculations revealed that the replacement of Fe^3+^ ions with Co^3+^ ions in octahedral positions leads to the modulation of O_2_ adsorption energy. Consequently, this alteration elongates the O–O bond, accelerates the activation of oxygen, facilitates the breaking of O–O bond, and increases ORR electrocatalytic activity [41] (Figure 3c).

## 3. Magnetic Structure of Spinel Ferrites: The Factors That Impact the Magnetic Properties

**Figure 4:**
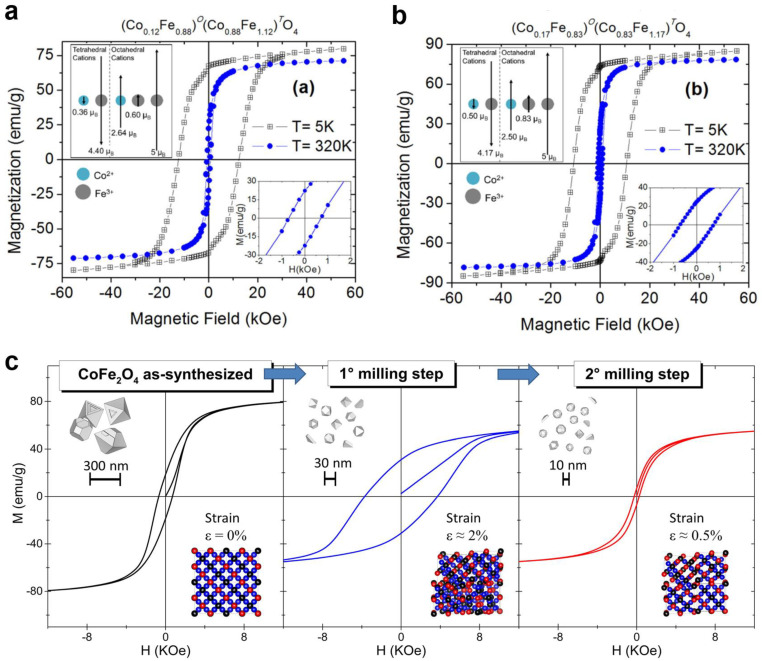
(**a**,**b**) M–H hysteresis loops, obtained at 5 and 320 K, for CoFe_2_O_4_ NPs synthesized by the coprecipitation method and calcined at (**a**) 873 K and (**b**) 1073 K (the upper insets show a scheme of the magnetic ordering between the A- and B-cations) (reprinted from [58], Copyright (2020), with permission from Sociedad Mexicana de Física, A.C). (**c**) M–H hysteresis loops for cobalt ferrite obtained via two-step planetary milling treatment (the insets in the right bottom corner show the level of strain induced by milling) (reprinted from [59], Copyright (2017), with permission from Elsevier).

Spinel ferrites, renowned for their distinct magnetic properties, have found widespread applications across a diverse range of industries, including electronics [43], electromagnetic wave absorption [28,44], high-frequency engineering [45,46], catalysis [41,47], biomedicine [48,49], chemical technology [50], and water purification [51,52,53,54]. The magnetic characteristics of spinel ferrites are contingent upon the intricacies of the crystal structure, specifically the cationic composition [55,56]. When the composition of spinel ferrite encompasses ions possessing individual magnetic moments, these moments orient themselves in a particular manner, giving rise to the magnetic structure of the spinel crystal [34,57]. For example, the M–H hysteresis loops observed at temperatures of 5 K and 320 K and depicted in Figure 4a,b exemplify the magnetic behavior of CoFe_2_O_4_ nanoparticles synthesized via the coprecipitation method and subjected to calcination at 873 K and 1073 K [58]. Evidently, the coercivity values measured at 5 K were approximately 12.4 kOe and 10.7 kOe for nanoparticles calcined at 873 K and 1073 K, respectively [58]. The disparities in M_S_ originated from the distinct cation distribution between the A- and B-sites within the spinel lattice. Moreover, the upper insets in Figure 4a,b schematically illustrate the magnetic ordering between Co^2+^ and Fe^3+^ cations at the A- and B-positions.

Increasing the coercivity can be achieved by elevating the defect density and residual strain of the nanoparticles via mechanical milling synthesis [59]. A two-step planetary milling treatment was implemented on cobalt ferrite to produce stressed and highly defective particles to investigate the impact of residual strain on their magnetic properties [59] (Figure 4c). The study’s findings indicated a correlation between magnetic properties and microstructure, revealing that the milling treatments led to the introduction of small magnetic defects. These defects exhibited a pinning effect on the magnetic domain wall motion, surpassing the impact of extended planar inhomogeneities such as twinning boundaries [59].

**Figure 5:**
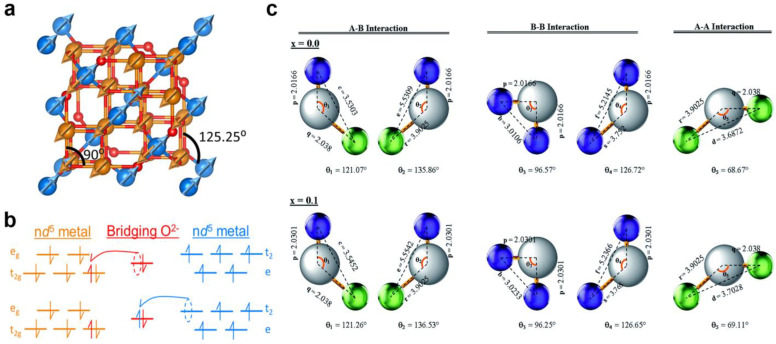
(**a**) The magnetic moments of the A-ions (blue) and the antiparallel magnetic moments of B-ions (orange) of MFe_2_O_4_ nanoparticles (M = Mn, Ni, and Zn) (reprinted with permission from [34]. Copyright 2021 American Chemical Society). (**b**) The scheme of super-exchange interaction, resulting in antiferromagnetic coupling of the B- (orange) and A- (blue) sites (reprinted with permission from [34]. Copyright 2021 American Chemical Society). (**c**) Configurations of ion pairs in spinel ferrites with favorable distances and angles for effective magnetic interactions in Co_0.7_Zn_0.3_Gd_x_Fe_2−x_O_4_ samples (reprinted with permission from [61]. Copyright 2018 The Royal Society of Chemistry).

The replacement of iron ions by non-magnetic ions significantly changes the magnetic structure [30]. Considering the bonding between the nearest neighbor pairs at the A- and B-sites through oxygen ions, a robust super-exchange interaction arises, characterized by an antiferromagnetic (AFM) nature [60]. There are three possible types of super-exchange interactions in the spinel ferrites: M_A_–O–M_B_, M_A_–O–M_A_, and M_B_–O–M_B_. The interactions involving M_A_–O–M_A_ and M_B_–O–M_B_ bonds occur over bond angles close to 90°, while the M_A_–O–M_B_ interactions occur over more linear bond angles, approximately 125° (Figure 5a) [34]. Goodenough’s theory states that the attainment of half-filled orbitals by the A and B cations results in the manifestation of antiferromagnetic alignment of electron spins (Figure 5b). Additionally, due to the robust A–O–B interaction, which holds paramount significance, the aggregate magnetization aligns with the magnetic moment of the octahedral positions. This occurrence arises from the fact that in the spinel structure, the number of occupied octahedral positions is twice that of the tetrahedral positions. For example, the possible types of magnetic interactions are presented in Figure 5c for Gd-substituted Co–Zn ferrites, showing that the M_A_–O–M_B_ exchange interaction mechanism is strengthened more than those of the M_A_–O–M_A_ and M_B_–O–M_B_ interactions, and this results in enhancing the saturation magnetization [61].

**Figure 6:**
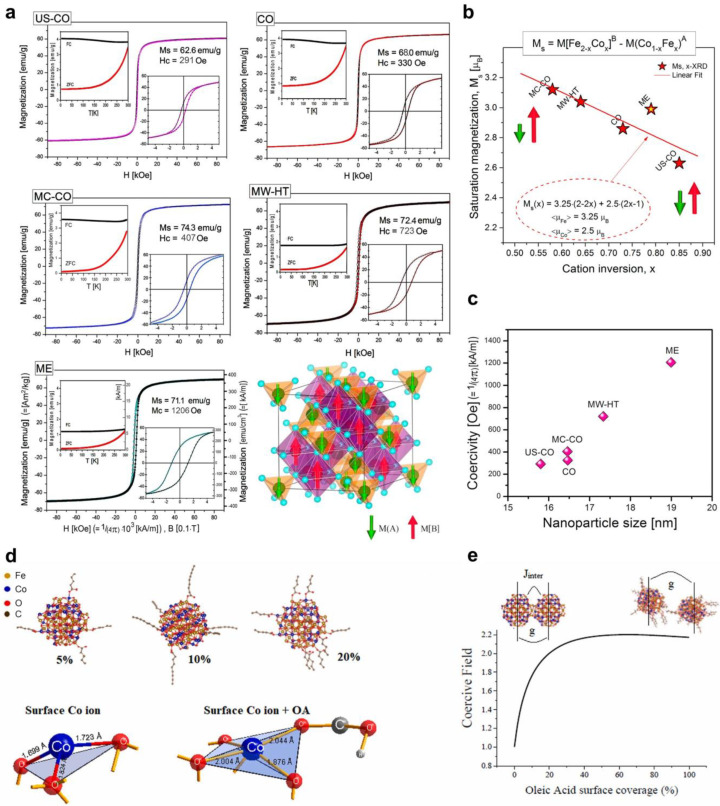
(**a**) Saturation magnetizations, hysteresis loops, and ZFC/FC measurements of cobalt ferrite NPs obtained by various synthesis methods. The ferrimagnetic spinel structure is depicted in the right-bottom corner (reprinted with permission from [62]. Copyright 2024 MDPI). (**b**) The saturation magnetization vs. cation inversion (x) (reprinted with permission from [62]. Copyright 2024 MDPI). (**c**) Coercivity vs. CoFe_2_O_4_ NPs size (reprinted with permission from [62]. Copyright 2024 MDPI). (**d**) Relaxed structures of CoFe_2_O_4_ NPs covered with different amounts of oleic acid and the surface Co ions without covering and bonded with OA in the CoFe_2_O_4_ structure (reprinted from [65], Copyright (2023), with permission from Elsevier). (**e**) Coercivity vs. oleic acid surface coverage onto cobalt ferrite NPs (reprinted from [65], Copyright (2023), with permission from Elsevier).

Milutinović et al. [62] established a reverse correlation between the degree of inversion of the spinel structure and the saturation magnetization. To achieve this, monodomain cobalt ferrite nanoparticles were synthesized through various methods, including coprecipitation (CO), ultrasonic-assisted coprecipitation (US-CO), coprecipitation with mechanochemical treatment (MC-CO), microemulsion route (ME), and microwave-assisted hydrothermal synthesis (MW-HT) [62]. The study revealed that the synthesis method significantly influences the saturation magnetization (Figure 6a), which, in turn, is determined by the degree of inversion (Figure 6b). Notably, the US-CO sample exhibited the highest inversion degree (x = 0.85) and a lowest saturation magnetization of 62.6 emu/g, while the MC-CO sample displayed the lowest inversion degree (x = 0.58) and a highest saturation magnetization of 74.3 emu/g. In addition, the coercivity, H_c_, increases with the size of the magnetic domains (Figure 6c). The conclusion is that not only the synthesis conditions but also the average NPs size, cation distribution, and NPs morphology crucially affect the magnetic properties and coercivity [62].

It is important to consider that the coercivity of magnetic nanoparticles can be influenced by the application of surface coatings [63,64,65]. For example, Vasilakaki et al. [65] conducted research on the modification of the magnetic properties of cobalt ferrite nanoparticles through the use of an oleic acid coating (Figure 6d). The findings revealed that using oleic acid molecules on the surface of cobalt ferrite NPs leads to a reduction in exchange interparticle energy, subsequently increasing coercivity (Figure 6e). Furthermore, when oleic acid molecules entirely cover the NPs surface, the exchange interparticle interactions are eliminated, leading to a decrease in coercivity [65].

Knowing how to design the distribution of defects and, accordingly, to influence the magnetic properties in a targeted manner is extremely important in the creation of magnetic spinel ferrite films for magneto-optical and spintronic devices [66,67]. For example, the mechanisms responsible for the magnetic order in zinc ferrite films and the impact of defect contribution on it are investigated in depth in the work [67]. The magnetic properties of spinel zinc ferrite thin films are found to be influenced by a multitude of factors, including the specific Zn-to-Fe ratio, the precise deposition conditions, the temperature during fabrication, and the type of gas atmosphere used [67]. These factors, in turn, are contingent upon the level of cation disorder and the precise distribution of defects within the thin film. The comprehensive analysis of these results reveals a compelling prospect: the deliberate engineering of cation distribution can potentially be harnessed to intricately tailor the magnetic properties of these films [67]. This finding holds considerable promise for the advancement of various device applications.

**Figure 7:**
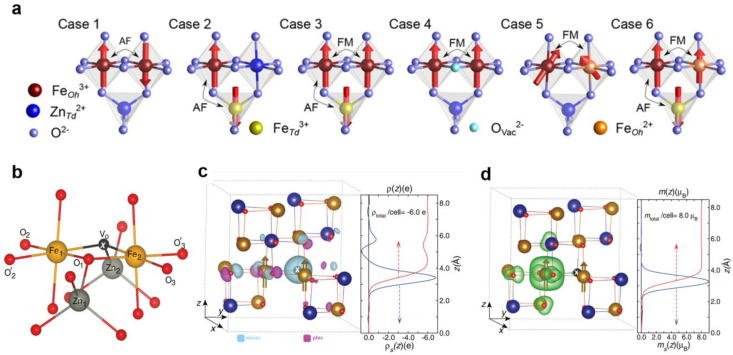
(**a**) The possible main magnetic configurations and interactions between the neighboring cations from ordered normal spinel ZnFe_2_O_4_ (Case 1) to inverse magnetite spinel Fe_3_O_4_ (Case 6). Case 4 is an example of disordered ZnFe_2_O_4_ with the presence of an oxygen vacancy (O_Vac_^2−^) (reprinted from [67], Copyright (2020), with permission from Wiley). (**b**) Local structure of ZnFe_2_O_4_ to explain the relaxation effect in the local environment of the oxygen vacancy (reprinted with permission from [71]. Copyright (2014) by the American Physical Society). (**c**,**d**) The isosurfaces and differences in (**c**) the charge density Δρ(r) and (**d**) the magnetization density Δm(r) around an oxygen vacancy (sphere with white cross) compared to the ideal structure (reprinted with permission from [71]. Copyright (2014) by the American Physical Society).

The magnetic properties of spinels are predominantly governed by indirect magnetic exchange interactions between iron ions [67,68,69,70]. The magnetic moment of Fe^2+^ ions is equivalent to 4 μB, while that of Fe^3+^ ions equals 5 μB. These interactions occur via mediation by oxygen anions, with the iron ions occupying tetra- and octahedral sites within the spinel structure. This arrangement results in ferromagnetic (FM) double-exchange (DE) and antiferromagnetic (AF) super-exchange (SE) interactions, ultimately contributing to the overall magnetic order (Figure 7a) [67]. As shown in Figure 7a (Case 4), the presence of oxygen vacancies strongly influences the net magnetic moment of ZnFe_2_O_4_ [67]. The lack of oxygen ions between the two Fe_B_ ions results not only in a change in bond length but also in the occurrence of ferromagnetic exchange interaction and the appearance of Fe^2+^ ions, attributable to the overall lattice charge neutrality [67]. Furthermore, the existence of oxygen vacancies induces a ferrimagnetic order in ZnFe_2_O_4_ at room temperature by affecting the neighboring atoms. In the work [71], it was established that the appearance of free space leads to the elongation of the Zn_1_–O_1_ bond by about 0.3% and part of the Fe_2_–O_1_ bonds by about 2.0%, as shown in Figure 7b [71]. The authors calculated the charge and magnetization densities (Figure 7c,d) in the spinel lattice with the presence of the oxygen vacancy. The charge density at the vacancy site significantly differs, leading to a distinct change in the magnetization density of the Fe atoms. This alteration results in the presence of ferromagnetic Fe_B_–Fe_B_ interactions, rather than the expected antiferromagnetic Fe_B_^3+^–O^2−^–Fe_B_^3+^ interactions. As a result, comprehending the effects of defects, specifically anion vacancies, becomes pivotal for a comprehensive analysis of the magnetic properties of spinel ferrites.

## 4. Defects in Spinels: Types and Notation

The extent of crystal lattice imperfection is contingent upon various factors such as temperature, environmental conditions, and the intrinsic properties of the material [72,73]. The occurrence of imperfections is intricately linked to the synthesis conditions, either stemming from kinetic causes or arising due to the introduction of impurities into the crystal [74]. Such imperfections can be categorized into two main groups: (i) atomic defects, resulting from the displacement of atoms/ions from their ideal positions within the crystal lattice, playing a pivotal role in characterizing the structural imperfections of the crystal in solid-state chemistry; and (ii) electronic defects, encompassing an excess of electrons and holes, as well as phenomena such as polarons and excitons, within the system [75].

**Figure 8:**
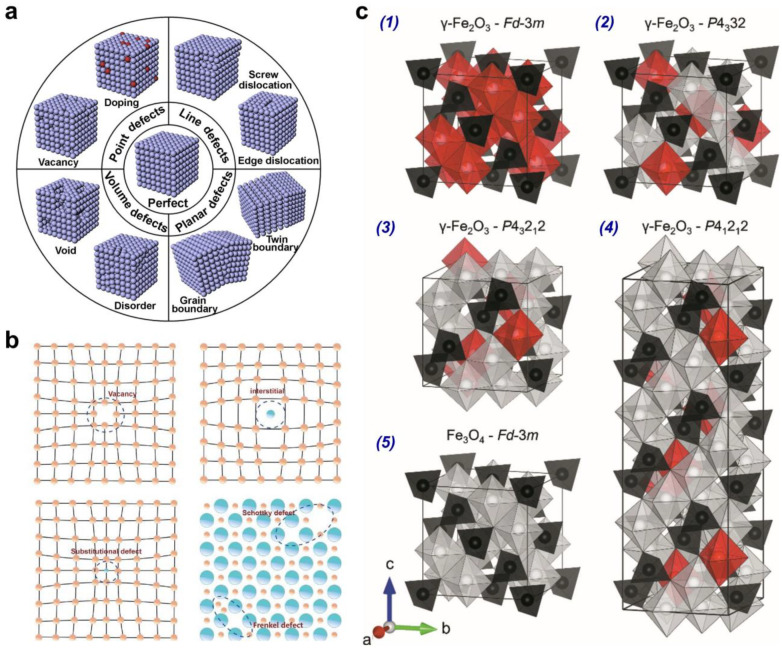
(**a**) The common classification of defects (reprinted from [7], Copyright (2018), with permission from Elsevier). (**b**) The types of point defects: vacancy, interstitial defect, substitutional defect, Frenkel defect, and Schottky defect (reprinted from [89], Copyright (2020), with permission from Elsevier). (**c**) Illustrations of the possible structural configurations (1)–(4) in the γ-Fe_2_O_3_ and the structure of Fe_3_O_4_ (5) (black polyhedra are A-sites, white polyhedra are B-sites, red polyhedra are partially occupied or unoccupied B-sites) (reprinted from [81], Copyright (2021), with permission from International Union of Crystallography).

There are several approaches to the classification of atomic defects [76]. According to stoichiometry, atomic defects are divided into stoichiometric and non-stoichiometric. When stoichiometric defects are formed, the chemical composition of compounds does not change. Nonstoichiometric defects are associated with changes in the chemical composition of substances [76]. From the point of view of chemical homogeneity, atomic defects in the crystal lattice can be divided into intrinsic and impurity defects. Impurity defects are caused by the presence of non-basic, additional atoms in the crystal structure. Impurity defects are also non-stoichiometric defects. According to the dimensionality, the defects are divided into point (0D), linear (1D), surface (2D), and bulk (3D) defects [7] (Figure 8a).

Point (0D) defects (Figure 8b) include vacancies or free nodes of the crystal lattice, atoms between nodes, and impurity atoms in the crystal lattice. The presence of point defects in ferrites can result in localized violations of charge electroneutrality, leading to electronic disorder. To be more precise, the point defects include Schottky defects, Frenkel defects, antisite defects, aliovalent substitutional defects, isovalent substitutional defects, and F-centers (F^2+^, F^+^, and F^0^) [77]. *F*-center is an anion vacancy, representing a deficit in negative charge and consequently serves as an electron trap, necessitating the capture of an electron to restore electroneutrality [78,79,80].

Vacancies are the most common type of atomic defect. Vacancies are the result of the transition of atoms from regular nodes to the crystal surface A_A_ + V_S_ = V_A_ + A_S_ or internodes A_A_ + V*_i_* = V_A_ + A*_i_*. In the first case, a single vacancy V_A_ is formed, and in the second case, a (V_A_ + A*_i_*) paired defect is formed. The formation of vacancies arises from the disparity between the actual energy of an individual atom and the average energy of all atoms within the system. At temperatures above 0 K, certain atoms within the crystal exhibit significantly higher energy than the majority of atoms. Consequently, some of these atoms possess adequate energy to depart from their regular lattice positions. The number of vacancies (n) formed in a simple crystal at a certain temperature (T) due to the transition of atoms to the crystal surface can be estimated using the following formula: $n_{v}=\alpha Ne^{\frac{-\Delta E_{v}}{kT}}$, where N is the total number of atoms in the crystal; $\Delta E_{v}$ is an energy of one vacancy formation; α is the coefficient that takes into account the entropy factor, $\alpha =e^{\Delta S/k}$; *k* is Boltzmann constant; T is temperature. The common example of a spinel compound with vacancy defects is maghemite γ-Fe_2_O_3_, which contains only trivalent Fe^3+^ ions (Figure 8c). Charge neutrality in this compound is maintained by introducing vacancies into B-sites [81]. The degree of vacancy ordering mostly depends on the synthesis route and thermal treatment.

Dislocations and chains of point defects can be attributed to linear (1D) defects (Figure 8a). The dislocations, in turn, are divided into edge and screw dislocations [82]. Surface (2D) or planar defects are crystal faces, boundaries of blocks, domains, grains, or impurity phases in crystals [83]. Antiphase domains are planar defects that occur in ordered solid solutions with crystal lattices formed as a superposition of two identical sublattices (for example, face-centered) that are superimposed on each other. Antiphase domains are bounded by antiphase boundaries (APBs) [29,84]. All chemical bonds and the arrangement of atoms correspond to the ideal structure inside the domain, while at the boundaries, there is a violation of the chemical bond. Such defects can be shared over the entire volume of ferrite films, worsening the electrical and optical properties, leading to a reduction in the piezoelectric coefficient and causing incomplete polarization in ferroelectrics [85,86]. Therefore, knowing the mechanisms and regularities of the formation of APBs defects, it is possible to prevent their occurrence. The twin boundary in crystals is a boundary between two regions of crystal, rotated at different angles relative to each other. The one region of the crystal differs from the other by a mirror reflection in the plane of symmetry (twin plane) or by rotation around the axis of symmetry (twin axis). If the boundary deviates from the twinning plane, it becomes irregular and contains twinning dislocations. Bulk (3D) defects are voids, inclusions of impurity phases, and other macroscopic formations in the crystals [87] (Figure 8a).

The most common approach for describing crystal defects is the Kröger–Vink notation [88]. According to it, a general uppercase letter indicates the type of defect (atom, vacancy, etc.), and the subscript indicates the crystallographic position of the defect (site, interstitials, etc.). For example, if a crystal consists only of atoms A, the defects have the following designation: V_A_ is a vacancy in the A site; A_i_ is an atom in a position between sites (interstitial atom); F_A_ is an impurity atom F in A site; V_i_ is an interstitial site; A_A_ is an atom A in a regular site A. Accordingly, the defects in the crystal of a binary AB compound are marked as follows: A_i_ and B_i_ are the interstitials atoms A and B; V_A_ and V_B_ are the vacancies in sublattices A and B, respectively; A_B_ is an atom A in the B site (antistructural defect); F_A_ (F_B,_ F_i_) is an impurity atom F in a site of the sublattice A (sublattice B or interstitial atom). Interstitial atoms (ions) are atoms (ions) occupying positions in the crystal structure that should be free in an ideal crystal.

The concept of relative charge is used to indicate the charge of defects. It is denoted as follows: • is a positive charge; ^/^ is a negative charge; × is a zero charge. Electrons and holes are denoted as e^/^and h^•^. The examples of some charged-defect designations are as follows: $V_{A}^{/}\;and\;V_{A}^{//}$ are the negatively charged vacancies (trapping of one or two electrons by a vacancy, respectively); $V_{B}^{•}\;and\;V_{B}^{••}$ are the positively charged vacancies (detachment of one or two electrons from vacancy, respectively); $A_{A}^{/}$ is an electron on an atom A; $B_{B}^{•}$ is a hole on an atom B (an absence of an electron); $V_{B}^{\times }$ is a neutral vacancy in the B sublattice. Antistructural defects are atoms (ions) located in places that are intended for other atoms (ions). They are denoted by A_B_, B_A_, B_C_, C_B_, etc. For example, in the case of a binary A_n_B_m_ crystal, the formation of two types of vacancies, V_A_ and V_B_, is feasible, along with two types of antistructural defects, A_B_ and B_A_.

The above-mentioned nomenclature of defects is convenient because it allows for describing charged defects in any crystal quite simply, regardless of the type of chemical bond and the real charge of atoms and ions in the crystal. Taking into account that the real charge of the ions/atoms in the crystal lattice in most cases cannot be determined, the system of relative charges of atoms in the crystal lattice becomes the only possible way to describe the real crystals.

## 5. The Impact of Defects on the Catalytic Properties of Ferrites

Ferrites are phases with variable chemical composition and different types of defects [90]. During the sintering, it is observed that oxygen atoms can permeate the environment from the surface layer of the ferrite [91,92]. Concomitantly, an excess of cations and anion vacancies emerges within the surface layer, exhibiting a propensity to uniformly disperse throughout the ferrite structure and permeate deeply into the material. This phenomenon results in a departure from the stoichiometric composition of the ferrites, leading to structural disorder [93]. The variety of disorder types can be notably extensive in ferrites containing multivalent ions. The primary cause of non-stoichiometry in ferrites is a modification in their chemical composition [94]. Additionally, other contributing factors to ferrite nonstoichiometry include variations in the electronic configurations of cations, the incorporation of elements with a different valence than the main ions, etc. [95,96]. The complete arrangement of vacancies or interstitial atoms (ions) at a specific concentration results in the development of a superstructure [97]. However, it is important to emphasize that there is currently no singular model that comprehensively describes the interaction of point defects, the formation of superstructures, and the organization and elimination of defects in spinels.

Plenty of papers are dedicated to the discernible influence of defects on the catalytic activity of ferrites [8,24,98,99,100]. It is evident that the spinel structure commonly exhibits two primary types of defects that significantly influence the physicochemical properties: cation (metal) vacancies and anion (oxygen) vacancies. Oxygen vacancies are the most studied defects in spinel materials, which affect their electronic structure and determine the activity in heterogeneous catalysis. The spinels, which contain cobalt (II) and iron (III) ions, attract much attention [101,102]. They exhibit high activity due to their ability to easily form surface oxygen vacancies, which could play the role of active centers in the different catalytic processes: catalysis [103,104,105], photocatalysis [106,107], electrocatalysis [108,109,110], etc. The number of oxygen vacancies can be artificially increased by introducing cations with a lower oxidation state and/or a smaller cation radius into the crystal lattice or during the thermal treatment.

It was investigated that Cu doping facilitates the formation of oxygen vacancy on the surface of spinel oxide Co_3_O_4_ [111]. This material can be used as a catalyst for carbonyl sulfur gas (COS) hydrolysis, which is contained in blast furnace gas and causes environmental pollution. It was shown that substitution with Cu in Co_3_O_4_ at level 10% (at.) drastically increases the surface basicity of the catalyst’s surface and promotes the formation of oxygen vacancies. Such defects are involved in the process of COS hydrolysis through the adsorption onto the 10Cu–Co_3_O_4_ catalyst. The mechanism consists of the activation of H_2_O molecules originating from the atmosphere, which compensates for the loss of surface hydroxyl groups and accelerates the reaction between them and COS [111]. The perspective approach to the design of bifunctional catalysts with enhanced adsorption activity through the formation of additional oxygen vacancies in spinel lattice can be used in environmental applications.

**Figure 9:**
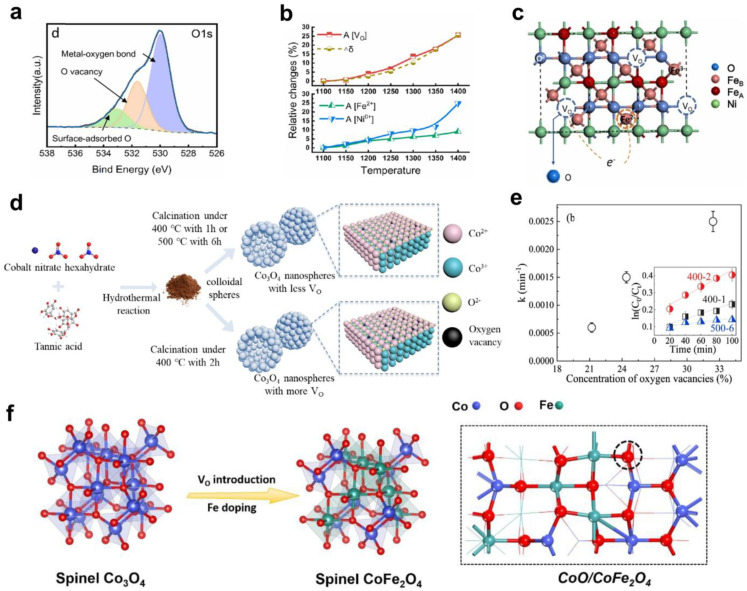
(**a**) The XPS spectra of O1s electrons in NiFe_2_O_4−δ_ sintered at 1300 °C (reprinted from [92], Copyright (2022), with permission from Elsevier); (**b**) Trends in the area ratios of the V_O_, Fe^2+^, and Ni^0^ peaks with sintering temperature in XPS fitting results (reprinted from [92], Copyright (2022), with permission from Elsevier); (**c**) Mechanism of oxygen vacancy formation in nickel ferrite lattice due to oxygen deficiency (reprinted from [92], Copyright (2022), with permission from Elsevier); (**d**) Hydrothermal synthesis of Co_3_O_4_ catalyst with oxygen vacancies (reprinted from [113], Copyright (2020), with permission from Elsevier); (**e**) Relationship between the amount of V_O_ and rate constant of PMS decomposition (reprinted from [113], Copyright (2020), with permission from Elsevier); (**f**) Scheme of the formation of defective CoO/CoFe_2_O_4_ material (dashed cycle is oxygen vacancy V_O_) (reprinted from [115], Copyright (2023), with permission from Elsevier).

Oxygen vacancies could appear during the sintering processes. It was investigated that the nonstoichiometry (oxygen deficiency) of nickel ferrite NiFe_2_O_4_ was increased during the sintering process and under oxygen partial pressures [92]. The rise in sintering temperature from 1150 to 1400 °C results in the rise of oxygen vacancy concentration in NiFe_2_O_4-δ_ (*δ* value increasing from 0.0014 to 0.0355), what was proved by Raman and XPS spectroscopies (Figure 4b). An increase in temperature also causes changes in the cation charge, promoting the formation of Fe^2+^ cations, which affects the formation of oxygen vacancies (Figure 9a–c) [92].

Oxygen vacancies (V_O_) in spinel-type catalysts are attracting much attention also due to their ability to decrease activation energy of reaction, facilitate the formation of singlet oxygen ^1^O_2_, and promote the movement of surface oxygen [108,112,113]. Moreover, the presence of V_O_ in catalyst structure could change the redox potentials of metals, accelerate the electron transfer between metal and oxygen species, and enhance the catalyst’s activity in radical-based advanced oxidation processes [114]. As it was shown in [113], the oxygen vacancy in spinel-type catalysts could play an essential role in the generation of singlet oxygen ^1^O_2_ during the PMS activation, which is important for environmental applications and water treatment. Spinel-type Co_2_MnO_4_ catalyst rich in V_O_ defects was synthesized by hydrothermal method (Figure 9d) and used to activate PMS in the phenol degradation. The relationship between the rate of PMS decomposition and the V_O_ concentration was investigated (Figure 9e): the higher the concentration of oxygen vacancies, the easier PMS molecules are adsorbed on the catalyst’s surface, which leads to lengthening and weakening of O–O bonds in the PMS molecules and their easier decomposition into active species (radicals) [113].

The presence of oxygen vacancies could enhance the electrocatalytic activity of spinel ferrites in the OER process. For example, the effects of oxygen vacancy engineering on the OER activity of bimetallic catalysts were investigated in [115]. The defective CoO/CoFe_2_O_4_ material was prepared by a technique that includes the solvothermal route and thermal treatment in a reduction atmosphere (using NaBH_4_). The formed interface in the proposed bimetallic catalyst contains a large amount of V_O_ and promotes an effective charge transfer, which is important for electrocatalytic applications (Figure 9f).

The cation vacancies also can affect the electronic structure, providing the formation of catalytically active sites [116]. Compared to oxygen vacancies, cation vacancies are more stable and can be easily formed during the synthesis. For example, the Mn-deficient Mn_3_O_4_ has been synthesized using solvothermal method [117]. The presence of Mn with different oxidation states (+2, +3, and +4) results in the formation of manganese vacancies which drastically improve the electrochemical reversibility of synthesized Mn–deficient Mn_3_O_4_ due to a reduction in the interfacial energy barrier for ion insertion and provide faster solid-state ion diffusion kinetics [117]. The cobalt-defected Co_3−x_O_4_ spinel was fabricated in situ by the solvothermal method and used as an OER catalyst [100]. The peculiarity of the proposed method is obtaining a glycerolatocobalt(II) precursor with a layered structure which promotes the formation of cobalt vacancies during thermal treatment. Metal vacancies cause the shifting of electronic density, which results in structure distortion, better structure stability, low corrosion rate, and long-term work in the water. In addition, Co-defected Co_3-x_O_4_ demonstrates a stronger affinity to bond water molecules for the OER process than normal Co_3_O_4_, resulting in its higher electrocatalytic activity [100].

## 6. Advanced Techniques for the Identification of Defects in Spinels

The identification of defects typically involves direct or indirect observations made through a combination of microscopic and/or spectroscopic techniques [2,26,118]. The microscopic methods could be used to characterize surface and bulk defects as well as the dissociated absorbed species on spinel surface and include transmission electron microscopy (TEM) [119], high-angle annular dark-field imaging in scanning transmission electron microscopy (HAADF-STEM) [98,120], high-resolution noncontact atomic force microscopy (NC-AFM) [121], light-element sensitive annular bright-field (ABF-STEM) [122], scanning tunneling microscopy (STM) [123], etc. Spectroscopic methods are used to characterize defects inside lattice structures, the chemical state of elements, and surface properties. Spectroscopic methods involve X-ray photoelectron spectroscopy (XPS) [124,125], positron annihilation lifetime spectroscopy (PALS) [99,126,127], Raman spectroscopy [128], Coincidence Doppler Broadening (CDB) spectroscopy [99], X-ray absorption spectroscopy (XAS) [98,129,130], etc. The analysis of the collected data leads to conclusions regarding defect types and the chemical surrounding the defects. X-ray photoelectron spectroscopy (XPS) took into account the changes in peak intensity, peaks shifting, or the appearance of new peaks, which could be the basis for conclusions about defect presence (in particular, vacancies). Synchrotron radiation-based X-ray absorption spectroscopy (XAS) is a powerful technique to investigate the local surroundings for each ion and its charge/electronic configuration. XAS includes X-ray absorption near-edge structure (XANES) and extended X-ray absorption fine structure (EXAFS). They intended to provide information about chemical as well as coordination environment in defective ferrites. The intensities of the pre-edge absorptions could serve as indicators of vacancy defects’ presence.

**Figure 10:**
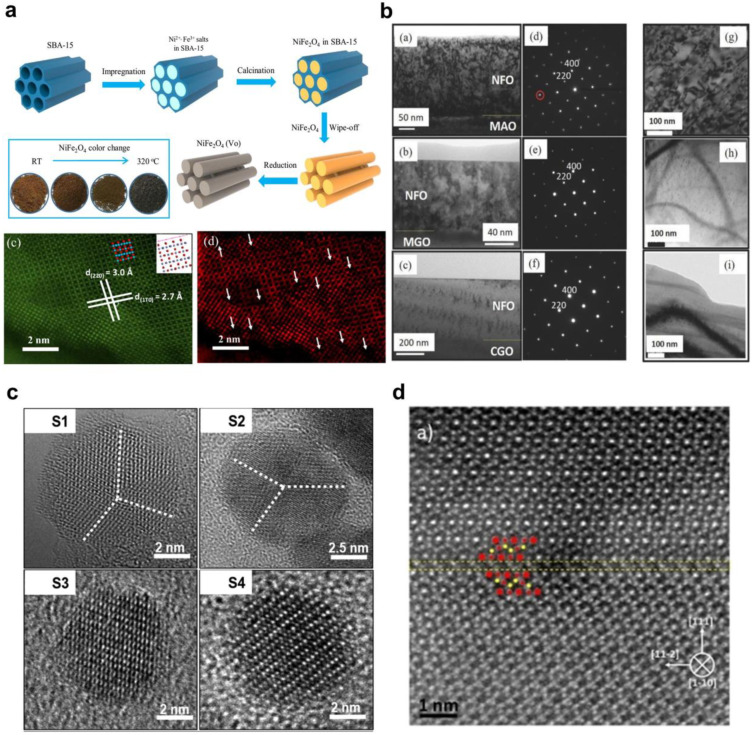
(**a**) A scheme of a one-step-impregnation hard-template method for obtaining mesoporous nickel ferrite. HAADF-STEM image of NiFe_2_O_4_ and live image simulated on GMS 3 software, with white arrows indicating oxygen vacancies in the NiFe_2_O_4_ structure (reprinted from [98], Copyright (2018), with permission from Elsevier). (**b**) TEM images and corresponding diffraction pattern taken for film/substrate for *(a,d)* MAO/NFO, *(b,e)* MGO/NFO, and *(c,f)* CGO/NFO thin films, respectively (separate spot shown by the red circle in *(d)* is due to strain relaxation); *(g)* diffraction contrast image of MAO/NFO shows the presence of APBs, whereas *(h)* MGO/NFO and *(i)* CGO/NFO do not show APBs defects (Copyright (2017) Wiley. Used with permission from [84]); (**c**) HRTEM images of cobalt ferrite NPs synthesized in the presence of different concentrations of 1,2-hexadecanediol as surfactant: S1 = 0 mM, S2 = 0.125 mM, S3 = 0.25 mM, and S4 = 0.5 mM (white dashed lines for S1 and S2 samples indicate crystallographic domain boundaries) (reprinted with permission from [119]. Copyright 2021 American Chemical Society); (**d**) HAADF-STEM image of the Fe_3_O_4_ with twin defect (outlined by yellow lines) with tetrahedral Fe_A_ sites in yellow and octahedral Fe_B_ sites in red (reprinted with permission from [120]. Copyright 2016 Springer Nature).

High-angle annular dark-field scanning transmission electron microscopy (HAADF-STEM) studies are crucial for identifying defects in crystalline structures and understanding the atomic-level structure of nanomaterials [98,131,132,133]. For example, Yue et al. [98] performed a HAADF-STEM analysis of highly ordered mesoporous NiFe_2_O_4_ synthesized using a one-step-impregnation hard-template method (Figure 10a). This synthesis successfully resulted in the formation of abundant oxygen vacancies in the mesoporous nickel ferrite, as evidenced by the HAADF-STEM technique (Figure 10a) [98]. These oxygen vacancies served as active sites for enhancing the catalytic activity of the material in the oxygen evolution reaction (OER) for water-splitting applications.

The HR-TEM is also the main method of observation of APBs defects. For example, the elimination of antiphase boundaries in epitaxial spinel NiFe_2_O_4_ (NFO) thin films was investigated in [84]. The growth was performed on three isostructural substrates MgAl_2_O_4_ (MAO), MgGa_2_O_4_ (MGO), and CoGa_2_O_4_ (CGO) using pulsed-laser deposition. The MAO substrates were acquired directly from CrysTec GmbH (Berlin, Germany). Single crystals of MGO and CGO, ranging from 1.5 to 2 cm in diameter, were grown utilizing the Czochralski method. MGO crystals were grown under a neutral or oxidizing atmosphere, while CGO was grown under a slightly reducing atmosphere. Subsequently, NFO films were deposited on MAO, MGO, and CGO substrates using pulsed-laser deposition. The deposition process involved a KrF laser with a wavelength of 248 nm, a laser fluence of 1.2–1.5 J cm^−2^, a repetition rate of 10 Hz, an oxygen pressure of 10 mTorr, and a substrate temperature of 700 °C [84]. TEM observations (Figure 10b) proved that the features of the crystal structure of substrates impact the APBs formation. The MAO substrate promoted the APB formation, while the MGO and CGO substrates prevented it. This is because the MgAl_2_O_4_ substrate and NiFe_2_O_4_ are non-isostructural materials, while MgGa_2_O_4_ and CoGa_2_O_4_ are isostructural and lattice-matched substrates for NiFe_2_O_4_ thin films.

The cation distribution of ferrites can affect structural disorders and depends on the synthesis conditions. For example, the impact of 1,2-hexadecanediol as a surfactant on the structural disorder of cobalt ferrite nanoparticles was investigated in [119]. The HR-TEM technique helped to establish that the absence and the low amount of 1,2-hexadecanediol (0.125 mM) cause the formation of NPs with crystallographic domain boundaries randomly oriented throughout the whole particle volume, whereas the high amount of 1,2-hexadecanediol (0.25 and 0.5 mM) promotes the formation of highly ordered NPs practically without crystallographic defects (Figure 10c).

A stable twin defect in Fe_3_O_4_ thin films was investigated in [120]. Such a defect breaks the translational symmetry of the spinel crystal cell and changes the angles and lengths of the bonds (Figure 10d). These structural changes impact the non-bulk super-exchange interaction in the boundary vicinity which, in turn, modifies the magnetic and electronic properties of magnetite thin film used for device applications [134,135].

**Figure 11:**
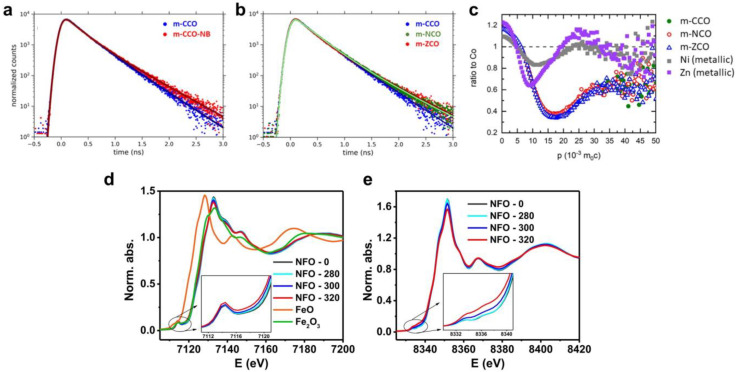
(**a**) Positron lifetime spectra for mesoporous CoCo_2_O_4_ and mesoporous NaBH_4_-treated CoCo_2_O_4_ samples (reprinted with permission from [99]. Copyright 2022 American Chemical Society). (**b**) Positron lifetime spectra for nontreated mesoporous samples: CoCo_2_O_4_, NiCo_2_O_4_, and ZnCo_2_O_4_ (reprinted with permission from [99]. Copyright 2022 American Chemical Society). (**c**) CDB ratio curves (related to pure metallic cobalt reference, dashed line) for nontreated CoCo_2_O_4_, NiCo_2_O_4_, and ZnCo_2_O_4_ samples (reprinted with permission from [99]. Copyright 2022 American Chemical Society). (**d**) Fe K-edge XANES spectra of mesoporous nickel ferrite (reprinted from [98], Copyright (2018), with permission from Elsevier). (**e**) Ni K-edge XANES spectra of mesoporous nickel ferrite (reprinted from [98], Copyright (2018), with permission from Elsevier).

Positron annihilation lifetime spectroscopy (PALS) is a technique used mostly for bulk material analysis and is an advanced characterization technique for analyzing the voids and defects in the crystal structure [99,127]. For example, Deeloed et al. used a PALS method to investigate the defects in defect-rich ordered mesoporous spinel oxides, including CoCo_2_O_4_, NiCo_2_O_4_, and ZnCo_2_O_4_, as well as CoCo_2_O_4_ treated with NaBH_4_ [99]. The PALS method involves measuring the lifetime of positrons in a spinel lattice. An increase in vacancies number in a sample leads to larger open volumes and consequently longer positron lifetimes. Initially, ab initio calculations of positron lifetimes were conducted using density-functional theory, involving the calculation of the lifetime of free positrons delocalized in the perfect lattice and positrons trapped at specific defect sites [99]. It was determined that the lifetime of free positrons delocalized in the perfect Co_3_O_4_ lattice is τ = 145.5 ps. The lifetime of positrons trapped by an oxygen vacancy is 147.8 ps, indicating that the vacancy is only a shallow trap. In contrast, cobalt vacancies are found to be much deeper traps. Considering the presence of two types of cobalt vacancies, the calculated lifetimes of positrons trapped in V_Co-1_ and V_Co-2_ were calculated at 174.8 and 217.6 ps, respectively. Furthermore, two types of vacancy pairs (V_Co_ + V_O_) were considered, with longer lifetimes of positrons calculated at 200.3 and 249.7 ps, respectively [99]. Figure 11a shows the positron lifetime spectra of the CoCo_2_O_4_ and NaBH_4_-treated CoCo_2_O_4_ samples. For both spectra, the lifetimes τ_1_ = 239 ps and τ_2_ = 242 ps are higher than the theoretical positron lifetime in a perfect Co_3_O_4_ lattice, τ_B_ = 145.5 ps [99]. Thus, a longer time is evidence of the presence of vacancies at the grain boundaries and indicates the increased concentration of vacancies after NaBH_4_ treatment. Figure 11b illustrates a comparison of positron lifetime spectra for all non-NaBH_4_-treated samples. It was observed that the positron lifetime for NiCo_2_O_4_ and ZnCo_2_O_4_ is significantly longer compared to the positron lifetime for the CoCo_2_O_4_ sample (τ_1_(NiCo_2_O_4_) = 283 ps, τ_1_(ZnCo_2_O_4_) = 253 ps). This suggests that both samples contain more surface vacancy defects with a larger open volume. Furthermore, the NaBH_4_ treatment noticeably increased the concentration of defects at the inter-grain boundaries [99].

Coincidence Doppler broadening (CDB) spectroscopy is a versatile tool for gathering information about the local chemical environment of defects. The presented Figure 11c displays CDB ratio curves for mesoporous CoCo_2_O_4_, NiCo_2_O_4_, and ZnCo_2_O_4_ samples [99]. Observation of Figure 11c reveals strikingly similar CDB ratio curves for non-substituted and Ni- and Zn-substituted samples, suggesting insignificant Ni(II) and Zn(II) segregation at open-volume defects. This indicates that neither the Ni(II) nor Zn(II) cations are bonded with these defect sites [99]. The authors [99] attribute the increased lifetimes τ_1_ and τ_2_ for NiCo_2_O_4_ and ZnCo_2_O_4_ samples to the transformation of vacancy pairs (V_Co_ + V_O_) into (V_Co_ + nV_O_ (2 ≤ n ≤ 4)). In essence, this implies an increased presence of oxygen vacancies at surface defect sites in Ni- and Zn-containing samples [99].

The present-day utilization of extended X-ray absorption fine structure (EXAFS) and X-ray absorption near edge structure (XANES) has increasingly been prevalent in conducting comprehensive examinations of ferrite structure and ascertaining the cation distribution in ferrite spinels [98,129]. EXAFS provides insights into bond distances and coordination numbers of shells enveloping the absorbing atom, whereas XANES furnishes details about the site symmetry and oxidation state of the absorbing atom [129]. Yue et al. [98] performed XAS studies for mesoporous nickel ferrite, synthesized by the one-step-impregnation hard-template method. Figure 11d,e demonstrate the Fe K-edge and Ni K-edge XANES spectra of mesoporous nickel ferrite [98]. The XANES spectrum provides valuable insights into the valence states of iron (Fe) and nickel (Ni) in comparison to a reference, based on edge absorption positions. At the Fe K-edge (Figure 11d), a distinct 1s→3d pre-edge transition feature at approximately 7114 eV is evident, attributed to disrupted octahedral symmetry resulting from cation vacancies. Upon subjecting nickel ferrites to increased thermal treatment temperatures, there is a corresponding rise in pre-edge absorption intensities, indicating an augmented concentration of oxygen vacancies. Similar observations are reported for the Ni K-edge XANES spectra (Figure 11e). Analysis of the structure parameters (specifically interatomic distance) derived from EXAFS data reveals the predominant localization of oxygen vacancies in proximity to nickel, attributable to the weaker Ni–O bond compared to the Fe–O bond [98].

## 7. Density-Functional Theory (DFT) for Studying the Point Defect Properties in Spinel Compounds

Advanced techniques can provide information about the presence of defects; however, these experiments often do not directly relate measurements to the charge, geometry, or type of defects. This necessitates the involvement of various simulation methods and software to complete the simulation process of defect formation in spinel materials. The most common simulation approach involves density-functional theory (DFT) that can simulate the potential pathways in the mechanisms of defect formation (e.g., vacancies, interstitials, or impurities) [136,137,138,139,140,141,142,143,144]. First-principles density-functional theory offers significant insights into defect identification, defect formation energies, and the neighbors of defects [136,137,138,139,140,141,142,143,144]. For example, Shi et al. [139] conducted first-principles calculations using DFT to examine the defect-free and defective bulk of NiCo_2_O_4_. The study investigated the impact of two defects, Ni ↔ Co(Td) exchanges, and oxygen vacancies, on the structural and electronic properties. It was observed that oxygen vacancies tend to occur primarily at sites coordinated with a higher number of Ni ions and are likely to form spontaneously at high temperatures and low oxygen pressures.

Shiiba et al. [140] employed ab initio DFT calculations to assess defect formation energies in disordered LiNi_0.5_Mn_1.5_O_4_ spinel, considering an oxygen vacancy model and a metal-excess model. The study concluded that the energy of defect formation for the metal-excess model is 2.58 eV, which is lower than that for the oxygen vacancy model, at 3.63 eV. This suggests that the formation of oxygen vacancies is not preferable, leading to the occupation of the octahedral vacancies by interstitial cations. Zasada et al. [141] investigated the structure and surface of cobalt spinel with various stoichiometry Co_3−x_O_4−y_ using spin-unrestricted DFT-PW91+U calculations. The study demonstrated that under reducing conditions (T > 600 °C and p_O2_/p° < 0.0001), the stoichiometric (100) facet surface becomes defective due to lattice oxygen release. In contrast, under oxidizing conditions (T < 200 °C and p_O2_/p° > 10), a coexistence of the (100) facets with stoichiometric and under-stoichiometric amounts of cobalt ions was observed. Furthermore, in a separate study by the same group, Zasada et al. [142] examined the (111) facet of cobalt spinel using periodic, spin-unrestricted DFT calculations, and first-principle thermodynamic modeling. The research evaluated the structure of nine different pristine and eight defected terminations of (111) surface of cobalt spinel with anionic and cationic vacancies under various redox conditions (T, p_O2_). The findings indicated that under more reducing conditions, the stoichiometric (111) surface allows for the reversible formation of oxygen surface vacancies, while under oxygen-rich conditions, cationic vacancies in the tetrahedral sites appear.

Bairagi et al. [136] proposed a potential pathway for the formation mechanism of spinel zinc aluminogallate. The researchers utilized density-functional theory within its generalized gradient approximation at the Perdew–Burke–Ernzerhof level of theory as implemented in the Quantum Espresso. The study involved modeling the structure and the energy costs for the substitution of Al and Ga atoms in the compounds. The analysis revealed that the presence of Ga vacancies reduces the formation-energy threshold, enabling interdiffusion and forming quaternary spinel zinc aluminogallate [136].

It is worth noting that DFT-based calculations of thermodynamic, diffusion, or optoelectronic properties in spinel materials, focusing on defects, are often constrained by limitations, time-consuming procedures, and relative complexity. This is primarily due to the reliance on periodic supercells used in DFT calculations of point defects, particularly defects’ concentration. Therefore, there is a critical need for advancements in automating these calculations [145]. However, despite this, density-functional theory is an important tool in materials science and engineering, offering valuable insights into the behavior of point defects.

## 8. Antistructural Mechanism of Defects Formation in Cobalt Ferrite through Dopant Ions Introduction

It is known that the crystal lattice’s imperfections determine the properties of solids, so the creation of defects allows for the creation of materials with predetermined properties. In this Section, the notation, called ‘antistructural notation’, for defect formation is presented for the first time for cobalt ferrite through dopant ions introduction. Antistructural modeling, a method used for forecasting, enables the estimation of the characteristics and concentration of intrinsic and impurity point defects, as well as the prediction of their impact on the physicochemical properties of ferrite compounds [146,147,148,149,150,151,152]. This modeling approach facilitates the description of active centers and their charge within the crystal lattice. The formation of point defects can be attributed to the introduction of donor and acceptor impurities, leading to the creation of localized electrons or holes within the structure. The presence of localized defects in a substance correlates with enhanced reactivity. For instance, introducing impurities into the crystal lattice can adjust the catalyst’s Fermi level position, consequently influencing its (photo)catalytic activity. In the antistructural notation proposed below, the incorporation of metal cations with varying charges (Cu(I), Mn(II), Ce(III), and Ce(IV)) into a spinel lattice is outlined.

**Figure 12:**
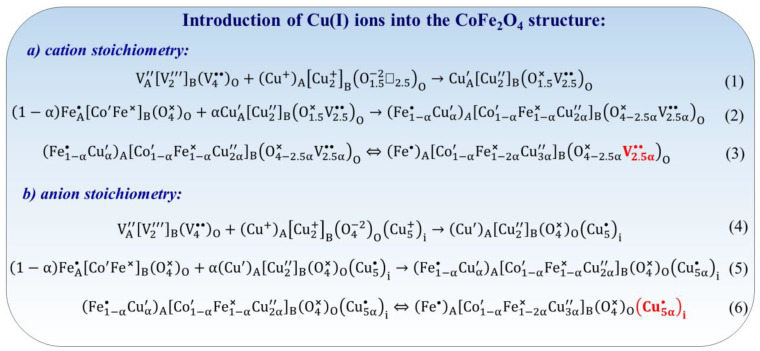
The antistructure mechanism of Cu(I) ions introduction into the CoFe_2_O_4_ structure (⎕ is an anion (oxygen) vacancy).

Introduction of Cu(I) into the CoFe_2_O_4_ structure: Antistructure modeling presents two potential avenues for mechanism formulation. The first is based on cation stoichiometry, while the second is based on anion stoichiometry (see Figure 12). Cation stoichiometry occurs when the quantity of cations in the dopant aligns with the number of cations in the matrix, as exemplified in the case of cobalt ferrite. For instance, the Cu_2_O oxide, serving as a source of Cu(I) ions, can be incorporated into the CoFe_2_O_4_ structure (Figure 12). Initially, the Cu_2_O must be transformed into the spinel structure by resonating the spinel antistructure with the crystallochemical structure of copper(I) oxide, as illustrated in Equation (1) in Figure 12. Cobalt ferrite may be considered as a crystalline matrix, with the oxide Cu_2_O serving as an impurity cluster that can be incorporated into the spinel matrix in varying quantities. In a general scenario, the cluster amount can be denoted as α mole, while ensuring that the sum of the matrix and cluster equals one mole. Consequently, the formulation of Cu-doped cobalt ferrite can be represented as illustrated in Equation (2) in Figure 12. Considering that Cu(I) ions exhibit a greater affinity for octahedral positions [153], whereas Fe(III) ions can occupy both tetrahedral and octahedral positions, Equation (2) in Figure 12 is transformed into Equation (3). This transformation gives rise to the observation of anion-vacancy defects and electronic defects. The maintenance of anions stoichiometry necessitates a correspondence between the number of oxygen ions in the oxide dopant and those in the matrix, exemplified by cobalt ferrite (Equations (4) and (5) in Figure 12). The heightened attraction of Cu(I) ions to octahedral positions instigated the conversion of Equation (5) to Equation (6) (Figure 12). Consequently, interstitial copper ions ${Cu}_{i}^{•}\;$ and copper ions with a surplus of negative charge in octahedral sites ${Cu}_{B}^{\prime \prime }$ are generated.

**Figure 13:**
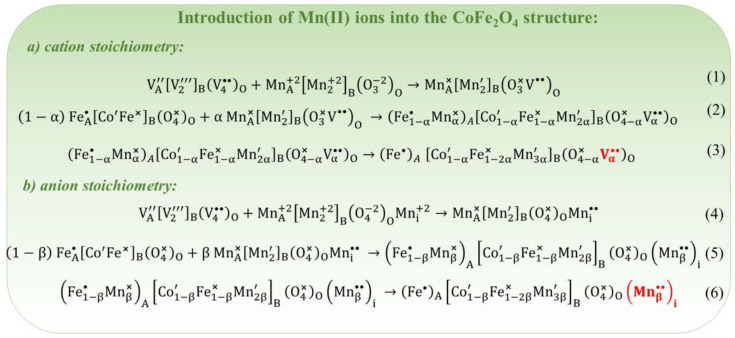
The antistructure mechanism of Mn(II) ions introduction into the CoFe_2_O_4_ structure.

Introduction of Mn(II) into the CoFe_2_O_4_ structure: As mentioned above, the prediction of defect formation can utilize two mechanisms based on cation stoichiometry and anion stoichiometry. Cation stoichiometry means that the quantity of Mn(II) ions in the dopant must align with the amount of metal cations in the matrix (cobalt ferrite). For instance, the conversion of MnO into the spinel structure can be achieved through the resonance of spinel antistructure with the crystallochemical structure of manganese(II) oxide (as shown in Equation (1) in Figure 13). MnO, an impurity cluster, can be incorporated into the cobalt ferrite matrix at a specified amount denoted as α mole. In contrast, the sum of the matrix and cluster should equal one mole. Consequently, the formation of the Mn-doped CoFe_2_O_4_ can be represented as Equation (2) in Figure 13. Considering that Mn^2+^ ions have a higher affinity for octahedral positions [154], Fe(III) ions can occupy both A- and B-positions, and Equation (2) should be transformed into Equation (3) (Figure 13). In this case, the anion-vacancy defects and electronic defects are observed. When the number of oxygen ions in the MnO oxide corresponds to the number of oxygen ions in the cobalt ferrite (anions stoichiometry), the antistructure mechanism is described by Equations (4) and (5) (Figure 13). Considering that Mn(II) ions have a higher affinity for octahedral positions [154], while Fe(III) ions can occupy both A- and B-positions, Equation (5) should be transformed into Equation (6) (Figure 13). This leads to the formation of interstitial manganese ions ${Mn}_{i}^{••}$ and manganese ions with an excess of negative charge in octahedral sites ${Mn}_{B}^{\prime \prime }$.

**Figure 14:**
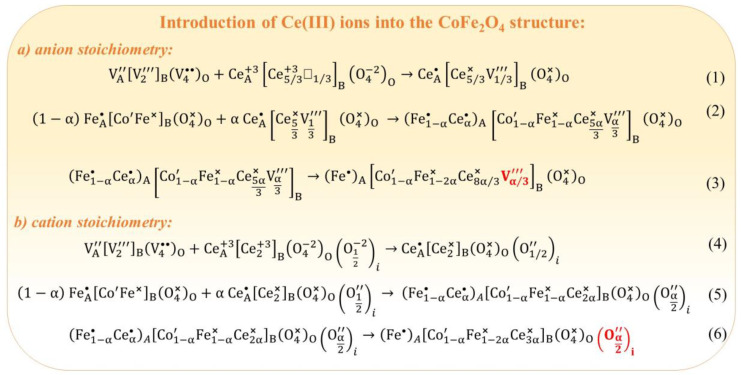
The antistructure mechanism of Ce(III) ions introduction into the CoFe_2_O_4_ structure (⎕ is an canion vacancy).

Introduction of Ce(III) into the CoFe_2_O_4_ structure: Ce(III) ions can be incorporated into the structure of cobalt spinel ferrite as an example of triple-charged ions. As mentioned above, both anion stoichiometry and cation stoichiometry are essential considerations. By considering Ce_2_O_3_ as a source of Ce(III) ions, the transformation of Ce_2_O_3_ into the spinel structure can be illustrated through the resonance of spinel antistructure with the crystallochemical structure of cerium(III) oxide (Equation (1) in Figure 14). Equations (2) and (3) in Figure 14 demonstrate potential pathways for the formation of cation vacancies in the B-sublattice. When considering cation stoichiometry, the conversion of 3/2 moles of Ce_2_O_3_ into the crystallochemical spinel formula can be described by Equation (4) (Figure 14). In this scenario, the formation of defects results in interstitial oxygen ions ${\left(O^{\prime \prime }\right)}_{i}$, as indicated by Equations (5) and (6) (Figure 14).

**Figure 15:**
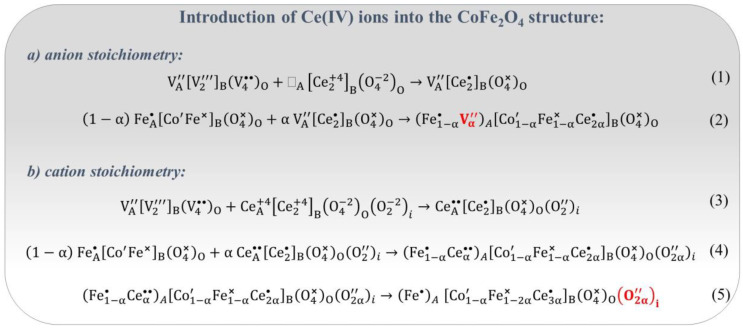
The antistructure mechanism of Ce(IV) ions introduction into the CoFe_2_O_4_ structure (⎕ is an canion vacancy).

Introduction of Ce(IV) into the CoFe_2_O_4_ structure. The incorporation of tetravalent Ce(IV) ions into the cobalt ferrite structure also induces lattice strains and the generation of point defects. Figure 15 illustrates two conceivable mechanisms for the introduction of Ce(IV) ion into CoFe_2_O_4_, based on anion or cation stoichiometry conditions. Equations (1) and (2) in Figure 15 outline the conversion of two moles of CeO_2_ into the spinel formula, creating the cation vacancies in the A-sublattice. Equations (3)–(5) in Figure 15 depict the conversion of three moles of CeO_2_ into the spinel structure owing to cation stoichiometry, followed by the generation of interstitial oxygen ions ${\left(O^{\prime \prime }\right)}_{i}$, potentially influencing the physicochemical properties of Ce-doped cobalt ferrite.

## 9. Defect-Engineering Strategies

**Figure 16:**
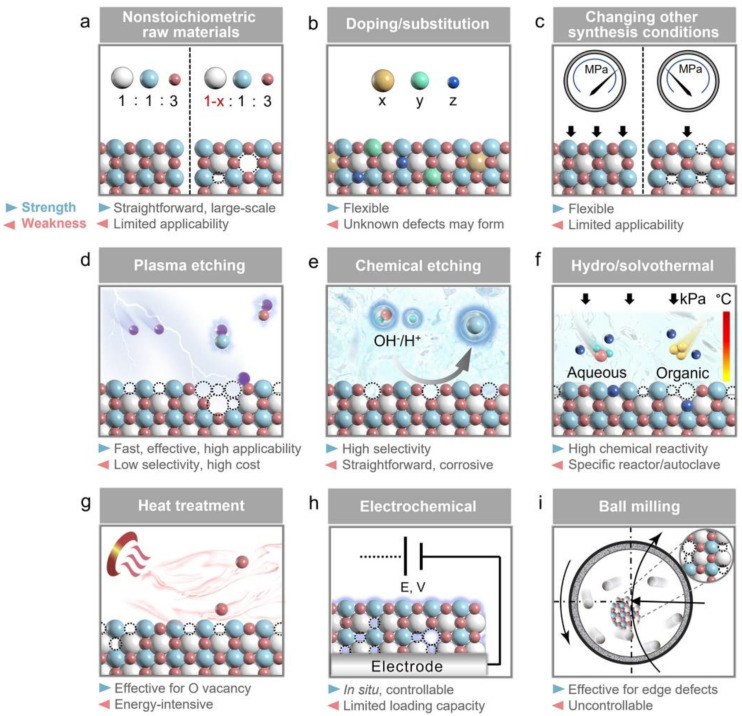
The strength (blue triangles) and weakness (red triangles) of different strategies used in defect engineering: (**a**) non-stoichiometric ratio between the precursors, (**b**) doping/substitution, (**c**) changing-synthesis conditions, (**d**) plasma etching, (**e**) chemical etching, (**f**) hydrothermal/solvothermal treatment, (**g**) heat treatment, (**h**) electrochemical treatment, and (**i**) ball milling (reprinted with permission from [137]. Copyright 2022 American Chemical Society).

In examining the influence of defects on the various properties of spinel compounds, it is essential to elucidate the relationship between defects and synthesis methods. In most cases, plenty of synthesis methods result in defects formation in spinel structure [30,155,156]. Generally, defect-engineering strategies can be categorized into two primary groups [137]: (I) the direct synthesis of defective spinel compounds and (II) the post-treatment of already synthesized spinel compounds to induce defects. The main strategies involved in defect engineering are illustrated in Figure 16 along with an assessment of the strengths and weaknesses of each approach [137]. These strategies include non-stoichiometric ratio between the precursors, doping/substitution, variations in pressure or temperature during synthesis, plasma etching, chemical etching, hydrothermal/solvothermal treatment, heat treatment, electrochemical treatment, and ball milling. It is noteworthy that certain strategies, such as doping/substitution, hydrothermal/solvothermal methods, and ball milling, can be applied in both direct synthesis and post-treatment processes [137]. As was mentioned, the most prevalent type of imperfections found in spinel ferrites are oxygen vacancies, which predominantly occur during the calcination and sintering processes, where the oxygen pressure and temperature exert significant influence. It is noteworthy that under low oxygen partial pressure, in the presence of nitrogen, or in a vacuum, the removal of oxygen from the spinel crystal lattice occurs, resulting in the formation of oxygen vacancies. Conversely, subjecting the spinel sample to heat treatment in the air leads to the replenishment of oxygen vacancies [155]. Table 1 presents the examples of spinel compounds along with the types of defects induced in them through various methods.

It is important to recognize that defect engineering remains a significant challenge in contemporary times. The deliberate creation of specific defects through certain methodologies presents difficulties. For instance, the selective extraction of particular atoms using high-energy techniques like ball milling or plasma etching poses a challenge. Conversely, softer approaches such as electrochemical treatment or the regulation of synthesis stoichiometry exhibit limited practicality [155].

## 10. Conclusions

Crystals commonly exhibit defects that significantly influence their physicochemical properties, encompassing mechanical, electrical, optical, catalytic, and adsorptive characteristics. The nature and prevalence of these defects are contingent upon the intrinsic attributes of the material, synthesis conditions, and the presence of impurities. This review meticulously examines the various defect types presented in spinel compounds, delving into an in-depth analysis of the magnetic structure and the determining factors affecting their magnetic properties. The review describes the structure, features, and properties of the most representative spinel compounds, e.g., CoFe_2_O_4_, NiFe_2_O_4_, ZnFe_2_O_4_, Fe_3_O_4_, γ–Fe_2_O_3_, Co_3_O_4_, Mn_3_O_4_, NiCo_2_O_4_, ZnCo_2_O_4_, Co_2_MnO_4_, etc. Attention was paid to the classification (0D, 1D, 2D, and 3D defects), nomenclature, and the formation of point and surface defects in ferrites. Comprehensive elucidation of the intricate relationship between defective structure and catalytic properties of materials is provided. Additionally, advanced microscopic and spectroscopic techniques, such as TEM, HAADF-STEM, STM, PALS, CDB, XANES, and EXAFS, are elucidated as they concern defect identification. The density-functional theory can be involved in the deep study of point defects in spinel compounds, showcasing its effectiveness in materials science and engineering. The relationship between defects formation and synthesis methods is described. Noteworthy emphasis is placed on antistructural notation as an innovative approach to delineate the mechanism of defect formation, particularly in instances involving the introduction of impurities into the crystal lattice of spinel ferrites. The antistructure mechanism shows that the introduction of ions into the spinel structure can lead to the formation of various point defects. This process can involve the substitution of ions at lattice sites, leading to the generation of vacancies, interstitials, or even anti-site defects. These defects can significantly impact the properties of the spinel material and are crucial in understanding its behavior and applications.

## Figures and Tables

**Table 1:** The examples of spinel compounds along with the types of defects induced in them through various methods.

Spinel Compound	Types of Defects	Method for Defects Creation	Application	Ref.
CuMn_2_O_4_	oxygen vacancies (V_O_), copper vacancies (V_Cu_), V_O_ combines with Mn_2_O_3_ at the interface between Mn_2_O_3_(222) and CuMn_2_O_4_(311))	alkali treatment	catalytic combustion	[157]
NiCo_2_O_4_	Co defects	plasma treatment	OER catalyst	[158]
ZnMn_2_O_4_	cation vacancies	hydrothermal method	cathode material	[159]
Li_x_Ni_1−2x_Fe_2+x_O_4_, x = 0, 0.05, 0.15, 0.25	oxygen vacancies	ball milling	hydroelectric cells	[160]
CoFe_2_O_4_	oxygen vacancies, defective domains	room-temperature lithium reduction strategy	OER catalyst	[83]
C-doped Fe_3_O_4_-based film	carbon interstitials and oxygen vacancies form carbon-oxygen vacancy (C_i_^B^–V_O_) pairs	low-temperature thermal decomposition method	spintronic devices	[66]
CoFe_2−x_O_4_	Fe vacancies	pulsed laser deposition	low-power electric-write and magnetic-read memories	[17]
Zn_x_Ni_1−x_Fe_2_O_4_, x = 0.2, 0.3, 0.4, and 0.5	oxygen vacancies	hydrothermal method	electrocatalyst for renewable hydrogen production	[161]
ZnFe_2_O_4_	oxygen vacancies	annealing under hydrogen flow and oxygen-poor conditions	photoelectrochemical water splitting	[162]
MnFe_2_O_4_	oxygen vacancies	sodium borohydride (NaBH_4_) calcination method	photocatalysis	[163]

## Data Availability

No new data were created or analyzed in this study. Data sharing is not applicable to this article.
